# The complete mitochondrial genome of *Hemiramphus far* (Beloniformes; Hemiramphidae) and phylogenetic studies of Beloniformes

**DOI:** 10.1080/23802359.2018.1532340

**Published:** 2018-10-25

**Authors:** Kehua Zhu, Zhenming Lü, Bingjian Liu, Li Gong, Lihua Jiang, Liqin Liu

**Affiliations:** a National Engineering Laboratory of Marine Germplasm Resources Exploration and Utilization, Zhejiang Ocean University, Zhoushan, China;; b National Engineering Research Center for Facilitated Marine Aquaculture, Marine science and technology college, Zhejiang Ocean University, Zhoushan, China

**Keywords:** Hemiramphus far, mitogenome, phylogenetic tree

## Abstract

The complete mitochondrial genome of this species was first determined in this study, which is 16,544 bp in length, the overall base composition includes C(27.0%), A(30.7%), T(27.1%) and G(15.2%). Moreover, the 13 protein-coding genes (PCGs) encode 3801 amino acids in total, 12 of which use the initiation codon ATG except COI uses GTG, most of them have TAA or TAG as the stop codon, whereas four protein-coding genes (COII, COIII, ND4 and Cytb) ended with the incomplete stop codon represented as a single T. The phylogenetic tree based on the neighbour-joining method was constructed to provide relationship within Beloniformes, the result supported that *H. far* was clustered with *Hyporhamphus dussumieri*.

*Hemiramphus far* is bathypelagic and is found in coastal waters of mountainous islands and continental shorelines, most frequently in areas of sea which are rich in vegetation and over sand flats (Shakman and Kinzelbach 2006). Talking about its economic value, *H. far* is commercially exploited along the Arabian Sea coast of Pakistan where the species is considered of great economic importance (Boughedir et al. 2015). Considering its ecological importance and commercial value, to gain its molecular information, we described the complete mitogenome of *H. far*, and explored the phylogenetic relationship within Beloniformes, which was expected to provide a new thought for management of this species.

The specimen was collected from South China Sea (16°49′12″N; 112°22′25″E) and stored in laboratory of Zhejiang Ocean University with accession number 20150826BZ22. Total genomic DNA was extracted from muscle tissue of individual using the phenol–chloroform method (Köchl et al. 2005). The calculation of base composition and phylogenetic construction was conducted by MEGA5.0 software (Tamura et al. 2011).

**Figure 1.**
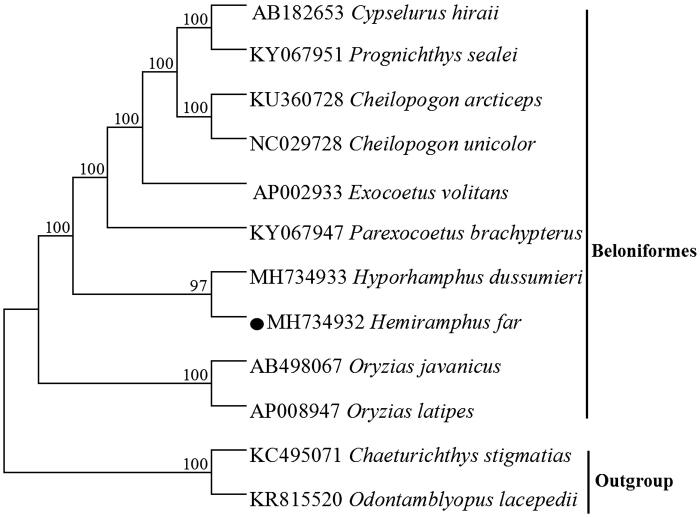
Neighbor Joining (NJ) tree of 10 Beloniformes species based on 12 PCGs encoded by the heavy strand. The bootstrap values are based on 1000 resamplings. The number at each node is the bootstrap probability. The number before the species name is the GenBank accession number. The genome sequence in this study is labeled with a black spot

Similar to the typical mitogenome of vertebrates, the mitogenome of *H. far* is a closed double-stranded circular molecule of 16,544 bp (GenBank accession no. MH734932), which contains 13 PCGs, two ribosomal RNA genes, 22 tRNA genes and two main non-coding regions. The overall base composition is 30.7%, 27.1%, 27.0% and 15.2% for A, T, C and G, respectively. Most mitochondrial genes are encoded on H-strand except for ND6 and eight tRNA genes (Gln, Ala, Asn, Cys, Tyr, Ser, Glu and Pro), which are encoded on the L-strand. Thirteen PCGs encode 3801 amino acids in total, all of them use the initiation codon ATG except COI uses GTG, which is quite common in vertebrate mtDNA (Zhu et al. 2018a, 2018b, 2018c). Four protein-coding genes (COII, COIII, ND4 and Cytb) were detected which ended with an incomplete stop codon T, these incomplete termination codons were presumably completed as TAA by post-transcriptional polyadenylation (Ojala et al. 1981). The 12S rRNA and 16S rRNA are 946 bp and 1692 bp, which are both located in the typical positions between tRNA-Phe and tRNA-Leu^(UUA)^, separated by tRNA-Val. The origin of light-strand replication is located in a cluster of five tRNA genes (WANCY) as in other vertebrates, and has the potential to fold into a stable stem-loop secondary structure, with a stem formed by 12 paired nucleotides and a loop of 12 nucleotides; The CR is determined to be 868 bp, by comparing the sequences of the CR with other teleost, three typical domains are observed, including termination-associated sequences, the central conserved sequence block domain and the conserved sequence block domain, which is identical to that in other teleostean mitogenomes (Zhang et al. 2013).

A phylogenetic tree was constructed based on the neighbour-joining method, the result supported that *H. far* was clustered with *Hyporhamphus dussumieri*, the similar result could be found in previous study (Tibbetts et al. 2007), which also provided essential and important DNA molecular data for further phylogenetic and evolutionary analysis for Beloniformes species.
